# Identification of Biomarkers for *Dendrobium officinale* Polysaccharide in Type 2 Diabetes Mellitus via Integrated Network Pharmacology and Mendelian Randomization

**DOI:** 10.3390/cimb48070672

**Published:** 2026-06-29

**Authors:** Yi Wu, Guimei Yang, Yixian Li, Yunjing Ruan, Qianmei Yang

**Affiliations:** 1 Experiment Center for Medical Science Research, Kunming Medical University, Kunming 650500, China; hearbler8651@126.com; 2 School of Pharmaceutical Science and Yunnan Provincial Key Laboratory of Pharmacology for Natural Products, Kunming Medical University, Kunming 650500, China; yangguimei@kmmu.edu.cn (G.Y.); lyx2282966@163.com (Y.L.); ruanyunjing806@163.com (Y.R.)

**Keywords:** type 2 diabetes mellitus, *Dendrobium officinale* polysaccharide, regulatory network, mendelian randomization, immune infiltration

## Abstract

*Dendrobium officinale* polysaccharide (DOP) shows efficacy against type 2 diabetes (T2D), but its mechanisms remain unclear. The present investigation aimed to identify potential biomarkers associated with DOP-mediated therapeutic interventions in T2D. Datasets related to T2D were excavated from the Gene Expression Omnibus (GEO) database. Candidate genes were acquired from the intersection of genes obtained from weighted gene co-expression network analysis (WGCNA) and differential analysis. Subsequently, Mendelian randomization (MR) identified causal biomarkers, validated by Receiver Operating Characteristic (ROC) curves and expression profiling. Then, a nomogram, immune infiltration, single-cell analysis, and molecular docking were performed. Among the 12 candidate genes, 7 with available eQTL instruments were included in MR analysis, while 5 lacking genome-wide significant IVs (*p* < 5 × 10^−8^) were excluded. Three genes demonstrated significant MR associations with T2D, and biomarkers *GLI1* and *LGALS9* showed strong diagnostic performance and were upregulated in T2D. The nomogram had good predictive value. Seventeen immune cells differed significantly between T2D and controls, with *GLI1* and *LGALS9* positively correlating with most and primarily expressed in stellate cells. Finally, D-Galacturonic acid, D-Mannose, and L-rhamnose monohydrate were compounds showing predicted binding potential with candidate biomarkers *GLI1* and *LGALS9* emerged as promising potential molecular candidates associated with DOP-mediated T2D regulation, offering novel mechanistic perspectives on DOP’s anti-diabetic properties.

## 1. Introduction

Type 2 diabetes mellitus (T2D) represents a multifaceted metabolic disease distinguished by sustained elevations in blood glucose levels, stemming from impaired insulin sensitivity coupled with gradual deterioration of pancreatic β-cell function. The global prevalence of T2D has increased dramatically over recent decades, posing a substantial burden on healthcare systems worldwide [1,2]. Chronic metabolic imbalance in T2D precipitates diverse complications encompassing cardiovascular pathologies, renal impairment, neurological disorders, and non-alcoholic fatty liver disease, consequently elevating both morbidity and mortality risks [3]. Although current diagnostic and therapeutic strategies have improved glycemic control, they remain insufficient to address disease heterogeneity and individual variability, highlighting the urgent need for reliable biomarkers and novel therapeutic targets.

In recent years, increasing attention has been paid to natural products as complementary or alternative therapeutic strategies for metabolic diseases. Owing to their multi-component and multi-target characteristics, natural compounds may modulate complex metabolic and inflammatory networks more effectively than single-target agents [4,5]. Dendrobium officinale, a traditional medicinal herb widely used in East Asia, has demonstrated various pharmacological activities, including anti-inflammatory, antioxidant, and hypoglycemic effects [6,7].

Across multiple animal models of T2D, *Dendrobium officinale* polysaccharides (DOP) exert glucose-lowering effects through diverse signaling pathways. DOP has been shown to act on the liver to ameliorate disturbances in glucose and lipid metabolism, partly by regulating key enzymes involved in hepatic gluconeogenesis and lipid metabolic processes [8]. More recent evidence further indicates that the antihyperglycemic actions of DOP are mediated through several complementary mechanisms. More specifically, DOP exerts beneficial effects through reshaping intestinal microbiota architecture, promoting short-chain fatty acid synthesis, and reinforcing gut barrier function, which collectively mitigate systemic inflammatory responses by downregulating the LPS/TLR4/TRIF/NF-κB signaling cascade [9]. In parallel, DOP has been reported to stimulate intestinal L-cell activity, promoting hormone secretion that enhances insulin release and improves glycemic control [10]. In addition, DOP may confer neuroprotective effects under diabetic conditions by preventing neuronal apoptosis through a TET2-dependent DNA demethylation mechanism, thus reducing the risk of diabetes-related neuropathic complications [11]. However, the core molecular targets underlying these effects remain unclear, particularly the DOP-responsive genes with causal relationships to T2D and their cell-type-specific regulatory roles. Given the structural complexity of polysaccharides, DOP may exert biological effects through both intact macromolecular structures and potential degradation products. Therefore, monosaccharide components were used as structural proxies in this study to facilitate computational target identification. This approach does not assume that free monosaccharides represent the exclusive active forms of DOP in vivo, but provides a systematic strategy to identify candidate molecular interactions for further validation. Systematic identification of the core target genes and clarification of their causal relationships are therefore essential for a deeper understanding of DOP’s antidiabetic mechanisms, as well as for the development of precise diagnostic biomarkers and the discovery of novel therapeutic targets.

Mendelian randomization (MR) leverages genetic variants as instrumental variables to infer causal relationships between gene expression and disease, thereby minimizing confounding bias and reverse causation inherent to observational studies [12]. However, MR analyses based on bulk tissue samples have limited ability to resolve gene functions within specific cellular contexts. Single-cell RNA sequencing (scRNA-seq), by contrast, enables dissection of gene expression heterogeneity at single-cell resolution and has provided critical evidence for cellular mechanisms in T2D [13,14]. The integration of MR and scRNA-seq therefore, offers a powerful approach to elucidate the cellular and molecular mechanisms underlying DOP’s therapeutic effects in T2D.

This study integrates transcriptomic data from the GEO database with predicted DOP targets, applying a multilayered bioinformatics framework to systematically identify core target genes through which DOP exerts therapeutic effects in T2D. By integrating differential gene expression profiling, co-expression network modeling, and MR-based causal inference, key biomarkers with causal links to T2D were identified, and their clinical diagnostic value was systematically assessed. These findings were complemented by immune microenvironment profiling, molecular docking simulations, single-cell transcriptomic analyses, and experimental validation. Collectively, this work delineates the molecular mechanisms of DOP-mediated intervention in T2D and provides insights for developing diagnostic markers and targeted therapeutic strategies.

## 2. Materials and Methods

### 2.1. Data Source

T2D-associated transcriptomic data were retrieved from the GEO database (https://www.ncbi.nlm.nih.gov/geo/; accessed on 6 December 2023), encompassing gene expression profiles from GSE164416 (GPL16791) and GSE25724 (GPL96) datasets, alongside single-cell RNA sequencing information derived from the GSE86469 cohort [15,16,17]. The GSE164416 dataset consisted of human pancreatic islet RNA-seq profiles from 133 donors, including 18 non-diabetic (ND), 41 impaired glucose tolerance (IGT), 35 type 3c diabetes (T3cD), and 39 T2D samples defined according to American Diabetes Association criteria. In this study, 39 T2D and 18 ND samples were selected as the training cohort. Gene IDs were converted from ENSEMBL IDs to gene symbols, and count data were normalized using the DESeq2 package. The validation cohort GSE25724 included 6 T2D and 7 control human pancreatic islet samples, and probe IDs were converted to gene symbols. Single-cell RNA sequencing data from GSE86469 included 638 human pancreatic islet-related cells from 5 non-diabetic and 3 T2D donors, and were used for cell-type-specific expression analysis. Since differential expression analysis and WGCNA were performed within the same bulk RNA-seq dataset (GSE164416), no cross-platform batch correction was applied. The monosaccharides of DOP were glucose, mannose, arabinose, galacturonic acid, xylose, rhamnose, and galactose. The SMILES or chemical structural formula of these monosaccharides was acquired from the PubChem database (https://pubchem.ncbi.nlm.nih.gov/; accessed on 6 December 2023). Target gene identification was subsequently conducted using the Swiss Target Prediction platform (http://swisstargetprediction.ch/, accessed on 6 December 2023), with species parameters configured for Homo sapiens. A total of 142 target genes for each monosaccharide were presented in Table S1 (Figure S1). The T2D genome-wide association study (GWAS) data were obtained from the DIAGRAM Consortium, encompassing a total of 74,124 European individuals diagnosed with T2D and 824,006 European control samples.

Expression quantitative trait locus (eQTL) GWAS datasets for candidate genes were extracted from the Integrative Epidemiology Unit (IEU) OpenGWAS repository (https://gwas.mrcieu.ac.uk/, accessed on 6 December 2023).

### 2.2. Differential Analysis

Differential gene expression analysis between T2D and control samples within the GSE164416 dataset was performed utilizing DESeq2 software (version 1.38.0), applying thresholds of |log2FC| > 0.5 and *p* < 0.05 to define T2D-differentially expressed genes (DEGs) [18]. Visualization was achieved through volcano plots generated via ggplot2 (version 3.4.1) and heatmaps constructed using ComplexHeatmap (version 2.16.0) [19,20].

### 2.3. Weighted Gene Co-Expression Network Analysis (WGCNA)

Utilizing the WGCNA package (version 1.71), the WGCNA of the gene expression matrix in samples within GSE164416 was carried out [21]. Initially, all samples underwent hierarchical clustering analysis with subsequent elimination of outlier specimens. Outlier samples were evaluated using the cutreeStatic function (cutHeight = 130, minSize = 10), and one sample was removed before downstream analysis. The optimal soft threshold parameter (β) was then established based on scale-free topology criteria. Afterwards, employing the optimal soft threshold as a basis, the module was segmented utilizing the standard of the hybrid dynamic tree cutting algorithm, and each module comprised a minimum of 100 genes. Finally, the modules exhibiting the strongest positive and negative correlation with T2D were selected as the pivotal models of T2D, and the T2D-related module genes were derived from these modules.

### 2.4. Identification and Function Enrichment Analysis of Candidate Genes, as Well as Protein–Protein Interaction (PPI) Network

The candidate genes were determined by the intersection between target genes, T2D-DEGs, and T2D-related module genes employing the ggvenn package (version 0.1.9) [22]. Functional enrichment analyses encompassing Gene Ontology (GO) annotations and Kyoto Encyclopedia of Genes and Genomes (KEGG) pathway mapping were conducted for candidate genes through clusterProfiler software (version 4.7.1.003), with statistical significance set at *p* < 0.05 [23]. PPI predictions among candidate gene products were generated via the STRING platform (https://string-db.org/; accessed on 6 December 2023), configured with Homo sapiens as the organism parameter and a minimal interaction confidence score of 0.4. Network visualization was subsequently achieved using Cytoscape software (version 3.7.1) [24].

### 2.5. Mendelian Randomization (MR) Analysis

MR analysis was employed with candidate genes serving as exposures and T2D designated as the outcome, aiming to identify biomarkers exhibiting causal associations with T2D. Three fundamental assumptions must be fulfilled in MR investigations: (a) instrumental variables (IVs) must demonstrate robust associations with exposure variables, (b) IVs should be independent of confounders linking exposures to outcomes, and (c) the influence of IVs on outcomes must be mediated exclusively through exposure pathways.

Initially, effect allele harmonization and effect size standardization were accomplished through the mv harmonise data function within TwoSampleMR (version 0.5.6) [12]. Subsequently, exposure data extraction and IV selection were executed utilizing VariantAnnotation (version 1.44.0) alongside ieugwasr (version 0.1.5), applying a genome-wide significance threshold of *p* < 5 × 10^–8^. This threshold effectively corrected for the multiple testing problem arising from millions of genetic variants across the genome, minimized the risk of false-positive associations between single nucleotide polymorphisms (SNPs) and exposure factors, and ensures the robustness of the association assumption in MR analysis.

To verify the independence assumption of MR analysis, namely that genetic IVs were unrelated to potential confounders of the exposure-outcome association, this study systematically assessed the associations between all included SNPs and predefined potential confounders of T2D.

Potential confounders were screened based on previous high-quality epidemiological studies on T2D, including body mass index (BMI), smoking status, alcohol consumption, hypertension, dyslipidemia, and fasting blood glucose levels. Phenotypic association data of each eligible SNP were retrieved from PhenoScanner V2 and the GWAS Catalog database, with the genome-wide significance threshold set at *p* < 5 × 10^–8^. Linkage disequilibrium (LD) clumping was performed to eliminate correlated IVs using parameters: clump = TRUE, R^2^ = 0.001, kb = 10 [25]. The IV was deemed reliable if the F value exceeded 10. Moreover, MR analysis of candidate genes was conducted by mr function combined with five algorithms (MR Egger, Weighted median, IVW, Simple mode, Weighted mode), of which the results of IVW were the primary reference (*p* < 0.05) [26,27,28,29,30]. The analytical findings were visually represented using scatter plots, forest plots, and funnel plots. An odds ratio (OR) greater than 1 indicated the association of the gene with an increased risk of T2D, whereas a value less than 1 suggested its potential as a protective factor.

### 2.6. The Sensitivity Analysis for MR Analysis and Steiger Directivity Analysis

MR result robustness was evaluated through comprehensive sensitivity analyses encompassing heterogeneity assessment, horizontal pleiotropy examination, and Leave-One-Out (LOO) validation. This study adopted a two-sample MR analysis to evaluate the causal effect of gene expression on the risk of T2D. The pre-specified statistical significance threshold for the primary analysis was set at *p* < 0.05. The IVW method was used as the core statistical approach to estimate the overall causal effect, which assumed that all included IVs were valid and free of horizontal pleiotropy. The fixed-effects IVW model was applied when no significant heterogeneity was observed (Cochran’s Q test *p* > 0.05). If significant heterogeneity existed with Cochran’s Q test *p* < 0.05, the random-effects model was adopted to correct for heterogeneity bias. Four complementary sensitivity analyses were performed to verify the robustness of the results: (1) Weighted median method, which can yield reliable causal estimates when more than 50% of IVs are valid; (2) Weighted mode method, which is insensitive to outlier IVs and horizontal pleiotropy; (3) LOO cross-validation, used to determine whether the overall findings were driven by any single outlier SNP; (4) MR-PRESSO analysis, employed to detect and correct outlier SNPs that cause horizontal pleiotropy. Cochran’s Q test was used to assess heterogeneity among IVs, and *p* < 0.05 was defined as statistically significant heterogeneity.

Horizontal pleiotropy was assessed using two complementary approaches: the MR-Egger regression intercept test (an intercept *p* > 0.05 indicates no significant horizontal pleiotropy) and the MR-PRESSO global test (a global test *p* > 0.05 suggests no overall significant horizontal pleiotropy). As this analysis comprised a pre-specified set of 7 candidate gene exposures selected through prior bioinformatic screening, conventional significance thresholds (*p* < 0.05) were applied without additional multiple-testing correction, consistent with hypothesis-driven candidate MR designs. Heterogeneity testing was initially conducted via the mr_heterogeneity function. Horizontal pleiotropy evaluation followed using the mr_pleiotropy_test function. Lastly, the LOO analysis was executed by the mr_leaveoneout function to assess if a solitary SNP could have a substantial impact on the overall outcomes. In addition, we performed a sensitivity analysis for IV selection using a more lenient *p*-value threshold of *p* < 1 × 10^–6^. Steiger directionality testing was performed using the Steiger_filtering function to verify the causal direction between exposure variables and outcome measures. A noteworthy *p* value (*p* < 0.05) and Steiger-Dir set to TRUE indicated that the direction was accurate. And the candidate genes exhibiting causal relationships with T2D were identified as potential biomarkers.

### 2.7. Receiver Operating Characteristic (ROC) Analysis and Creation of a Nomogram

In order to investigate the diagnostic potential of potential biomarkers, false positive and true positive values were computed by the pROC package (version 1.18.0) based on potential biomarkers expression data and disease classification information from GSE164416 and GSE25724 datasets, and ROC curves were generated to visualize the results [31]. Meanwhile, potential biomarkers exhibiting area under the curve (AUC) values greater than 0.7 in both datasets served as reliable biomarkers. The expression levels of biomarkers were subsequently validated in the above two datasets. Then, the rms package (version 6.5–0) was employed to fabricate a diagnostic nomogram for T2D patients according to biomarker expression [32]. Calibration curves and decision curve analysis (DCA) were constructed by rms package (version 6.5–0) and the ggDCA package (version 1.2), respectively, to assess the accuracy and reliability of the nomogram predictions [32]. Two independent GEO cohorts [GSE20966 (*n* = 10 T2D, *n* = 10 controls) and GSE38642 (*n* = 9 T2D, *n* = 54 controls)] were used for external validation.

### 2.8. Gene Set Enrichment Analysis (GSEA)

To elucidate biological pathways associated with identified biomarkers, Spearman correlation coefficients between individual biomarkers and the complete transcriptome were computed within the GSE164416 dataset, followed by gene ranking in descending order based on correlation strength. Gene Set Enrichment Analysis (GSEA) was subsequently executed using clusterProfiler (version 4.7.1.003) with KEGG gene collections (c2.cp.kegg.v2023.1.Hs.symbols.gmt) as reference, applying an adjusted *p*-value threshold of <0.05 [23]. The KEGG gene sets were obtained from the Molecular Signatures Database (MsigDB) repository (https://www.gsea-msigdb.org/gsea/msigdb; accessed on 6 December 2023).

### 2.9. Immune Infiltration Analysis

The single sample GSEA (ssGSEA) algorithm in the GSVA package (version 1.46.0) was adopted to evaluate the prevalence of 28 immune cells in both T2D and control groups [33]. The infiltration difference of 28 immune cells was analyzed by wilcox.test (*p* < 0.05). Furthermore, the Spearman correlation analysis was employed to calculate the associations among distinct immune cells and between biomarkers and these cells (*p* < 0.05).

### 2.10. Construction of Regulatory Network and Molecular Docking

To elucidate regulatory mechanisms governing biomarker expression, transcription factor (TF) prediction was initially performed through the JASPAR repository accessed via the NetworkAnalyst interface (https://www.networkanalyst.ca; accessed on 6 December 2023). Subsequent identification of miRNAs targeting these biomarkers was accomplished using the miRTarBase v8.0 database integrated within NetworkAnalyst. Integration of these regulatory elements into a comprehensive TF-mRNA-miRNA interaction network was visualized through Cytoscape software (version 3.7.1) [24]. According to the target drugs of biomarkers in 2.1, the PubChem database (https://pubchem.ncbi.nlm.nih.gov/; accessed on 6 December 2023) was adopted to obtain the three-dimensional structures of these drugs. Subsequently, protein crystal structures corresponding to biomarkers were obtained from the PDB database (https://www.rcsb.org/; accessed on 6 December 2023). Finally, molecular docking was performed using the CB-Dock database (http://cao.labshare.cn:10380/cb-dock2/php/manual.php; accessed on 6 December 2023). The docking scores were interpreted as indicators of potential molecular interactions rather than direct evidence of biological activity or therapeutic efficacy. To validate docking reliability, GANT61, a well-characterized small-molecule inhibitor of *GLI1* with documented binding activity, was used as a positive control [34].

### 2.11. Single-Cell RNA-Seq Analysis

Firstly, the Seurat package (version 4.3.0) was utilized for cell quality control in the GSE86469 dataset [35] cells with less than 200 genes and genes expressed in less than 3 cells were filter out, cells that possess several nFeature_RNA greater than 5000 and less than 10,000, as well as a number of nCount_RNA less than 2,000,000, were retained. After applying the NormalizeData function from the Seurat package (version 4.3.0) for data normalization, the subsequent step involved utilizing the FindVariableFeatures function to identify genes with high variability based on their mean-variance relationship [35]. Subsequently, the ScaleData function was implemented for data normalization, and the ElbowPlot function was employed to draw the elbow plot and select the principal components (PCs) in the PC analysis (PCA). The JackStraw function was utilized to compute the *p*-value of each gene in every PC for assessing its statistical significance. The ScoreJackStraw function was employed to quantify the strength of significance associated with each PC. Next, the uniform manifold approximation and projection (UMAP) cell cluster analysis was carried out applying the Seurat standard procedure (resolution = 0.4). Cell annotation was then performed based on literature-derived marker genes specific to each cell type [13]. Finally, the comparison was made between the expressions of biomarkers in cells of the T2D and control groups. Furthermore, the cell communication network was inferred after creating CellChat objects, importing the CellChatDB.human database (https://github.com/sqjin/CellChat; accessed on 6 December 2023), performing preprocessing, and conducting other necessary operations by CellChat package (version 1.6.1) [36]. The pseudotime analysis of annotated cells was simulated utilizing Monocle package (version 2.26.0) [37]. Then, the expressions of biomarkers in cells in pseudotime were analysed.

### 2.12. Statistical Analysis

The data was processed and analyzed using the R software (version 4.2.1). A significance level of *p* < 0.05 was deemed statistically significant.

## 3. Results

### 3.1. Acquisition of Candidate Genes

**Figure 1:**
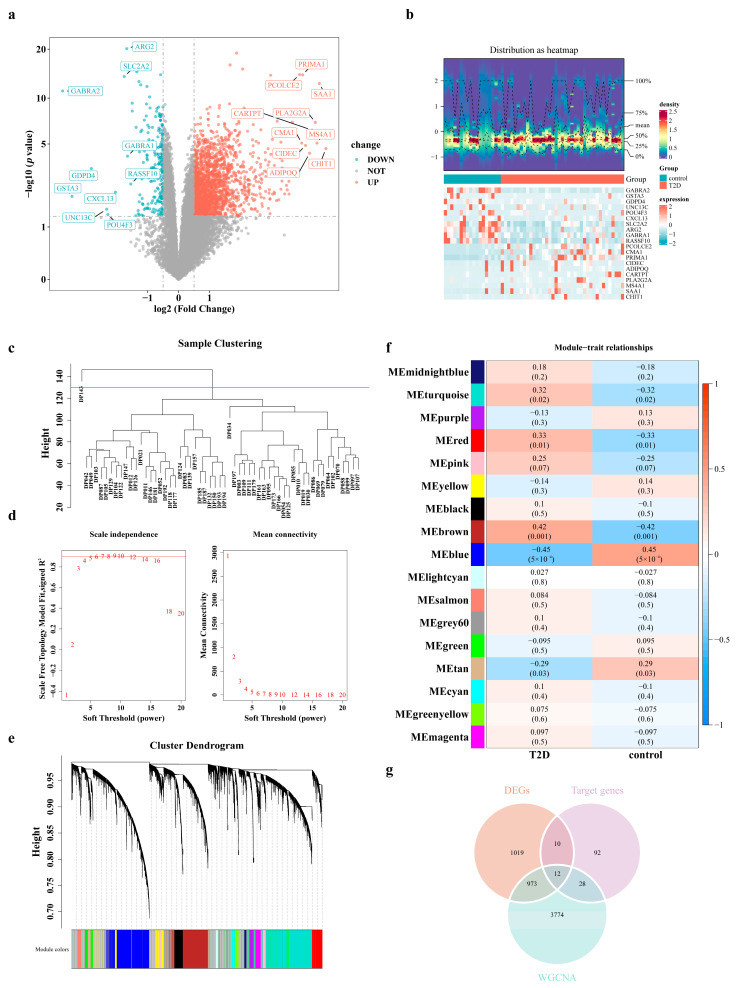
Identification of candidate genes through integrated transcriptomic and network analyses. (**a**) Volcano plot of differentially expressed genes (DEGs) in type 2 diabetes mellitus (T2D) in the GSE164416 dataset, with red dots representing significantly up-regulated genes and blue dots representing significantly down-regulated genes. (**b**) Heatmap of DEGs, where red indicates high expression and green indicates low expression. (**c**) Hierarchical clustering dendrogram of samples in the GSE164416 dataset. The red horizontal line marks the height threshold for sample clustering cutoff. (**d**) Selection of the soft threshold (β), where a value of 7 was chosen as it exceeded the red cut-off line. Red horizontal lines mark scale-free topology R^2^ cutoff and soft-threshold power; dendrogram dashed line denotes cluster cut height. (**e**) Clustering dendrogram of gene co-expression modules, with 18 distinct modules identified by the dynamic tree cut method and labeled with unique colors. (**f**) Module-trait correlation analysis, with red indicating positive correlation and blue indicating negative correlation. (**g**) Venn diagram illustrating the identification of candidate genes. Abbreviations: T2D, type 2 diabetes mellitus; DEGs, differentially expressed genes; WGCNA, weighted gene co-expression network analysis; DOP, *Dendrobium officinale* polysaccharide; FC, fold change.

Firstly, a total of 2014 T2D-DEGs were acquired by the difference analysis between T2D and control samples in GSE164416. Among these T2D-DEGs, 1812 were found to be up-regulated in T2D, while 202 were down-regulated. And the top 10 up-regulated T2D-DEGs and the top 10 down-regulated T2D-DEGs sequenced according to log2FC were illustrated by the volcano map and heat map (Figure 1a,b).

Subsequently, a sample clustering analysis was conducted on the GSE164416 dataset, leading to the identification and exclusion of DP143 as an outlier (Figure 1c). The value of β was established as 7 by selecting a threshold of scale-free R^2^ at 0.9 for the formation of gene modules (Figure 1d). Consequently, application of the hybrid dynamic tree cutting algorithm generated 18 distinct modules (Figure 1e). Among these, the brown module demonstrated maximal positive association with T2D (R = 0.42), whereas the blue module exhibited the most pronounced negative association (R = −0.45), collectively encompassing 4787 genes within T2D-related modules (Figure 1f). Ultimately, the identification of 12 candidate genes were based on overlap of 142 target genes, 2014 T2D-DEGs and 4787 T2D module genes (Figure 1g).

### 3.2. The Candidate Genes Were Significantly Enriched in Tertiary Granule, Insulin Resistance, Meningioma, and Gap Junction, Etc.

**Figure 2:**
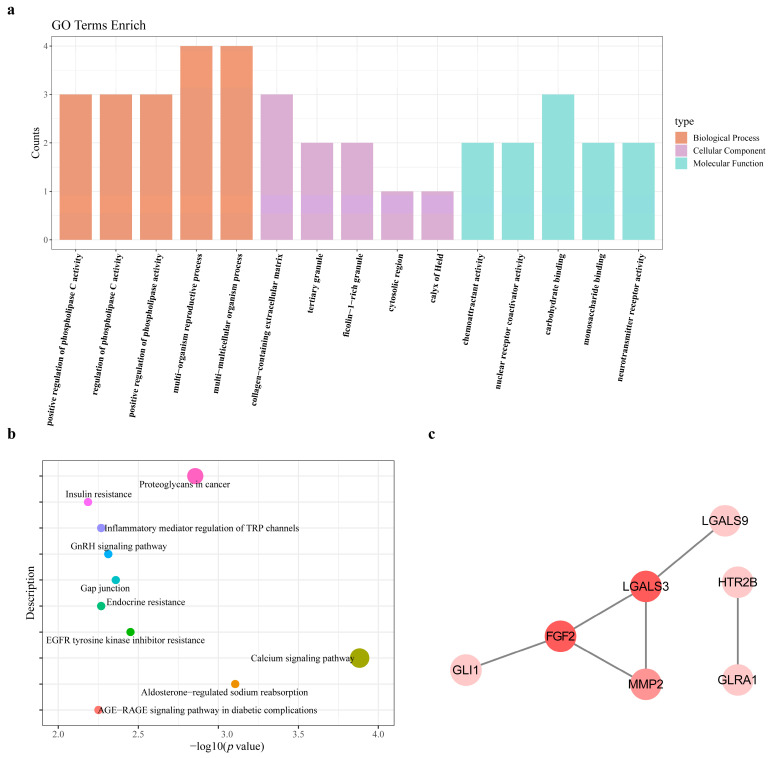
Functional analysis of candidate genes. (**a**) Bar plot of Gene Ontology (GO) functional enrichment analysis for candidate genes. Different colors represent distinct GO term categories; bar length indicates the number of genes enriched in each term. (**b**) Bubble plot of Kyoto Encyclopedia of Genes and Genomes (KEGG) pathway enrichment analysis for candidate genes. Larger circle size represents a greater number of genes enriched in the corresponding pathway. (**c**) Protein–protein interaction (PPI) network of candidate genes. Abbreviations: GO, Gene Ontology; KEGG, Kyoto Encyclopedia of Genes and Genomes; PPI, protein–protein interaction.

GO functional annotation of candidate genes revealed enrichment in biological processes including phospholipase C activity modulation (both positive regulation and general regulation), chemoattractant activity, tertiary granule formation, carbohydrate binding capacity, among others (Figure 2a). KEGG pathway analysis demonstrated significant enrichment within multiple pathways, notably cancer-associated proteoglycans, insulin resistance mechanisms, GnRH signaling cascade, gap junction communication, and additional relevant pathways (Figure 2b).

Furthermore, after removing the discrete proteins, a PPI network containing 7 genes was constructed to study the interactions between these genes. There were interactions among *FGF2, MMP2*, and *LGALS3*, while *HTR2B* interacted with *GCRA1* (Figure 2c).

### 3.3. Identification of Potential Biomarkers That Exhibited Noteworthy Causal Relationships with T2D

The exposure factors for the MR analysis consisted of a set of 12 candidate genes, out of which 7 candidate genes had SNPs, and the outcome used in this study was T2D. A total of 3 potential biomarkers (*GLI1*, *LGALS9*, and *FGF2*) were found to have notable causal relationships with T2D (*p* < 0.05 in IVW). *LGALS9* and *FGF2* exhibited odds ratios exceeding unity (OR = 1.011 and OR = 1.193, respectively), signifying their roles as risk-conferring factors in T2D pathogenesis. On the other hand, *GLI1* (OR = 0.928) had an odds ratio less than 1, suggesting it is a protective factor against T2D (Table S2).

Scatter plot and forest plot visualizations revealed positive associations between *LGALS9* and *FGF2* SNPs and T2D susceptibility, characterized by MR effect estimates greater than zero. Conversely, *GLI1* SNPs demonstrated inverse associations with T2D risk, reflected by MR effect estimates below zero. These findings corroborated the aforementioned MR analytical conclusions (Figures S2 and S3). Finally, funnel plot assessment confirmed that the MR examination of three candidate biomarkers in relation to T2D conformed to Mendelian randomization principles, consistent with the second law of independent assortment (Figure S4).

The sensitivity analysis outcomes indicated that there were no potential biomarkers with horizontal pleiotropy and heterogeneity. Additionally, the robustness and reliability of our MR analysis were confirmed through LOO analysis. (Tables S3 and S4, Figure S5). The results of Steiger directivity analysis showed that the direction of the three exposure factors and the outcome were correct (Table S5). All included SNPs showed no genome-wide significant associations with any of the predefined potential confounders, satisfying the independence assumption for MR analysis. In addition, the causal effect estimates derived from this sensitivity analysis were consistent in direction and statistical conclusion with those of the primary analysis, further confirming that the conclusions of this study are not biased by the selection of different *p*-value thresholds for IV screening. It confirmed that the conclusions of this study were not confounded by potential confounding factors.

### 3.4. GLI1, and LGALS9 Served as Dependable Biomarkers

**Figure 3:**
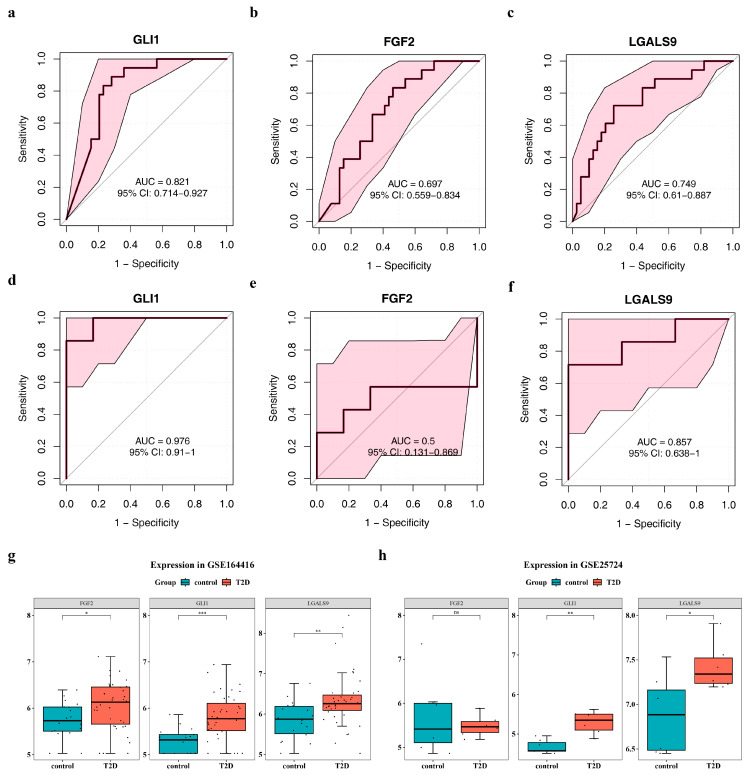
Identification of biomarkers through ROC analysis and expression validation. (**a**–**c**) Receiver operating characteristic (ROC) curve analysis of potential biomarkers in the GSE164416 dataset. (**d**–**f**) ROC curve analysis of potential biomarkers in the GSE25724 dataset. (**g**) Comparison of expression levels of potential biomarkers between type 2 diabetes mellitus (T2D) samples and control groups in the GSE164416 dataset. * *p* < 0.05, ** *p* < 0.01, *** *p* < 0.001. (**h**) Comparison of expression levels of *GLI1* and *LGALS9* between T2D samples and control groups in the GSE25724 dataset. * *p* < 0.05, ** *p* < 0.01, and ns indicates no significance. Abbreviations: ROC, receiver operating characteristic; AUC, area under the curve; T2D, type 2 diabetes mellitus.

**Figure 4:**
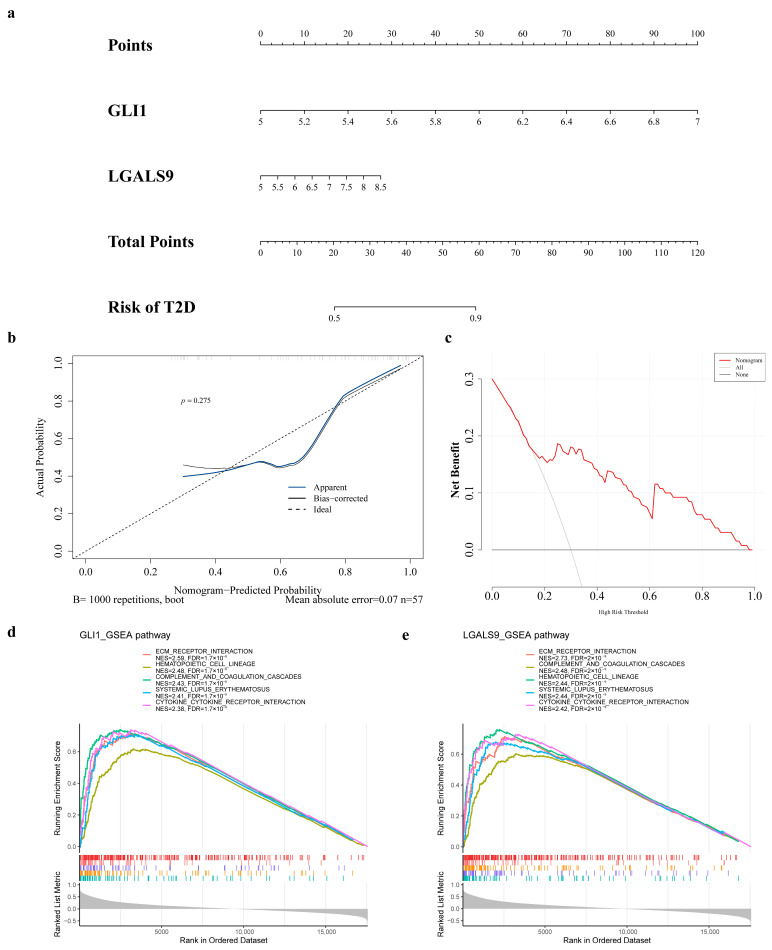
Construction of nomogram and functional pathway enrichment analysis of biomarkers. (**a**) Nomogram for predicting the probability of T2D based on *GLI1* and *LGALS9* expression levels in the GSE164416 dataset. Points represent the score for each variable. Calculate the total points of the biomarkers and then map them to the linear score and outcome probability. (**b**) Calibration curve of the nomogram. The predicted probability of the nomogram showed no significant difference from the reference line (*p* value > 0.05), indicating high predictive accuracy of the nomogram. (**c**) Decision curve analysis (DCA) of the nomogram model. (**d**,**e**) Gene Set Enrichment Analysis (GSEA) of *GLI1* (**d**) and *LGALS9* (**e**). Lines of different colors represent different pathways.

ROC analysis revealed AUC values of 0.821 (95% CI: 0.714–0.927) for *GLI1* and 0.749 (95% CI: 0.61–0.887) for *LGALS9* within the GSE164416 dataset, while the GSE25724 cohort demonstrated AUC values of 0.976 (95% CI: 0.91–1) and 0.857 (95% CI: 0.638–1) for these respective genes, indicating robust diagnostic potential as reliable biomarkers (Figure 3a–f). Both *GLI1* and *LGALS9* exhibited markedly increased transcriptional levels in T2D specimens across the GSE164416 and GSE25724 datasets (Figure 3g,h). Then. based on *GLI1* and *LGALS9*, a nomogram was constructed to predict the probability of developing T2D (Figure 4a). The prediction probability of the nomogram was not significantly different from that of the reference line in calibration curve and the benefit rate of the nomogram model (red line) was significantly higher than that of the slash line (All) and horizontal line (None) in DCA curve, which indicated a good predictive performance of the nomogram (Figure 4b,c). GSEA enrichment analysis demonstrated that *GLI1* and *LGALS9* were associated with extracellular matrix (ECM) receptor interaction, proteasome, and protein export, etc. (Figure 4d,e). ROC analysis was further performed in two independent cohorts, GSE20966 (*n* = 10 T2D vs. 10 controls) and GSE38642 (*n* = 9 T2D vs. 54 controls). In GSE20966, AUCs were 0.615 (95% CI: 0.350–0.880) for *GLI1* and 0.550 (95% CI: 0.277–0.823) for *LGALS9*; in GSE38642, AUCs were 0.595 (95% CI: 0.413–0.776) and 0.498 (95% CI: 0.312–0.684), respectively (Figure S6a–d). These findings suggest modest external diagnostic performance, with wide confidence intervals indicating uncertainty; larger independent cohorts are required for validation. To investigate the discrepancy between *GLI1* genetic protection and its upregulation in T2D, Spearman analysis was performed in GSE164416. *GLI1* expression was positively correlated with key insulin signaling genes, including *PIK3R1* and *IRS1*, suggesting that *GLI1* upregulation may represent a compensatory response to impaired insulin signaling rather than a pathogenic change (Figure S6e).

### 3.5. The Immune Infiltration Displayed Significant Disparities Between the T2D and Control Cohorts

**Figure 5:**
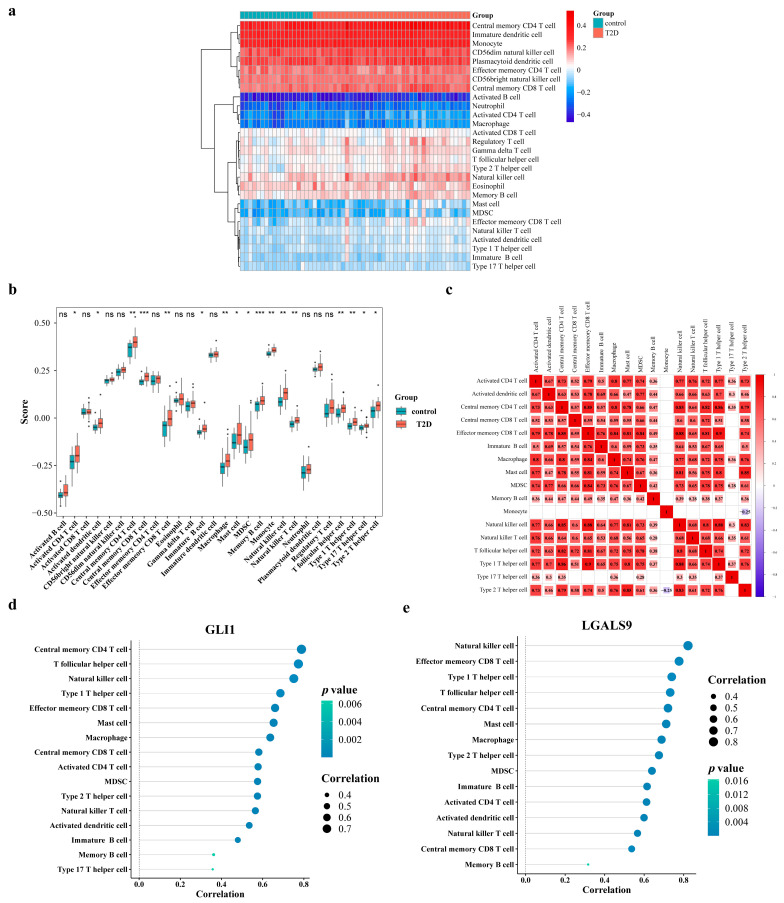
Immune infiltration analysis. (**a**) Heatmap showing the scores of 28 types of immune cells in type 2 diabetes mellitus (T2D) and control samples from the GSE164416 dataset. Red indicates high scores and blue indicates low scores. (**b**) Box plots showing the infiltration levels of immune cells with significant differences between the two groups. * *p* < 0.05, ** *p* < 0.01, *** *p* < 0.001, and ns indicates no significance. (**c**) Correlation heatmap of differentially infiltrated immune cells. Red indicates positive correlation and purple indicates negative correlation. (**d**,**e**) Correlation analysis results between *GLI1* (**d**) and *LGALS9* (**e**) with differentially infiltrated immune cells. Colors closer to blue indicate greater significance. Abbreviations: T2D, type 2 diabetes mellitus; ssGSEA, single-sample gene set enrichment analysis; NK, natural killer.

The heat map was utilized to visualize the distribution of 28 immune cells in both T2D and control groups within the samples (Figure 5a). 17 immune cells were existing notable disparities in these two groups, such as memory B cells, central memory CD8+ T cells, and activated CD4+ T cells (Figure 5b). Notably, the correlation between all differential immune cells was positive, except macrophages and type 2 helper T cells (Figure 5c). *GLI1* transcriptional levels demonstrated positive associations with 16 distinct immune cell populations, with central memory CD4+ T cells displaying the most pronounced correlation (cor = 0.79, *p* < 0.05). Comparably, *LGALS9* expression correlated positively with 15 immune cell subsets, achieving maximal correlation strength with natural killer (NK) cells (cor = 0.82, *p* < 0.05) (Figure 5d,e). It should be noted that ssGSEA results from bulk islet data reflect transcriptional enrichment rather than true immune infiltration and may be influenced by composition, inflammation, or batch effects; thus, they are hypothesis-generating only.

### 3.6. D-Galacturonic Acid, D-Mannose, and L-Rhamnose Monohydrate Showed Predicted Binding Potential with Identified Biomarkers

**Figure 6:**
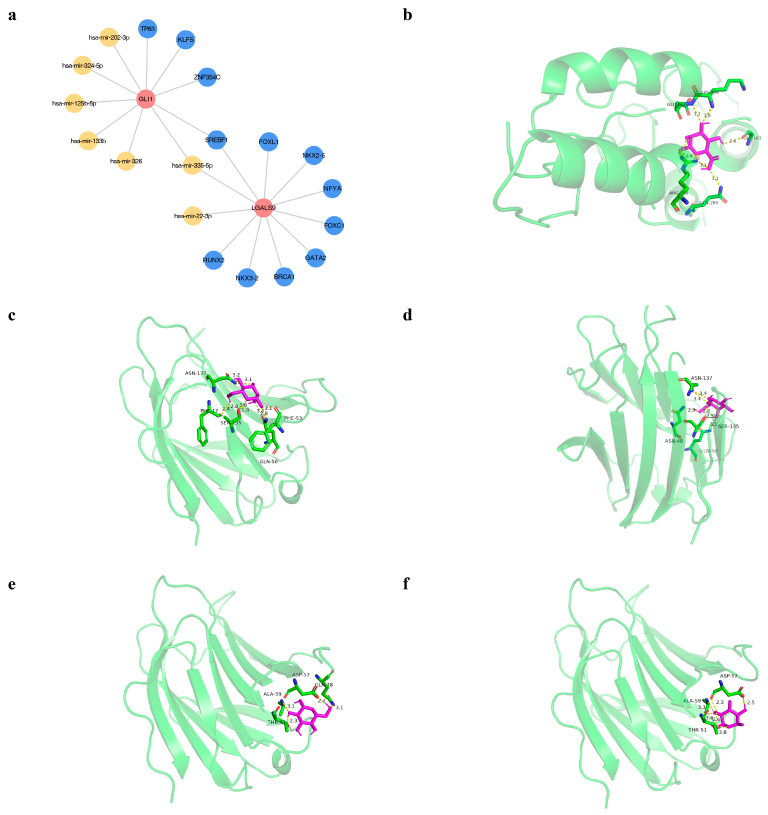
Construction of biomarker regulatory network and molecular docking. (**a**) TF/miRNA–biomarker network. Red represents biomarkers, blue represents transcription factors (TFs), and yellow represents miRNAs. (**b**) Docking model of *GLI1* with D-Galacturonic acid. (**c**) Docking model of *LGALS9* with D-Mannose. (**d**) Docking model of *LGALS9* with Galactose. (**e**) Docking model of *LGALS9* with glucose. (**f**) Docking model of *LGALS9* with L-rhamnose monohydrate. For (**b**–**f**) light green ribbons show the protein backbone; sticks with varied colors represent ligand structures; dotted lines stand for hydrogen bonds formed between amino acid residues and ligands. Abbreviations: TF, transcription factor; miRNA, microRNA; PDB, Protein Data Bank.

The network consisted of 12 transcription factors (TFs), 2 biomarkers, and 7 microRNAs, totaling 21 nodes and 21 edges (Figure 6a). The association between hsa-mir-335a-5p and *SREBF1* with both biomarkers suggested their crucial role in the mechanism of biomarker action in T2D. The target drug of *GLI1* was D-Galacturonic acid, and for *LGALS9*, the target drugs were glucose, D-Mannose, Galactose, and L-rhamnose monohydrate. Furthermore, the binding energy of *GLI1* and D-Galacturonic acid, as well as *LGALS9* with D-Mannose and L-rhamnose monohydrate, was less than −5, suggesting favorable predicted binding affinity. These results provide structural hypotheses for further experimental validation (Table 1, Figure 6b–f). To validate the docking protocol, GANT61 (a *GLI1* inhibitor) was docked to *GLI1* as a positive control, yielding a binding energy of −5.2 kcal/mol consistent with reported activity, confirming docking reliability. Interactions showed hydrogen bonds and key residue contacts visualized by PyMOL (v2.6; Figure S7).

### 3.7. Biomarkers Were Predominantly Expressed in Stellate Cells

**Figure 7:**
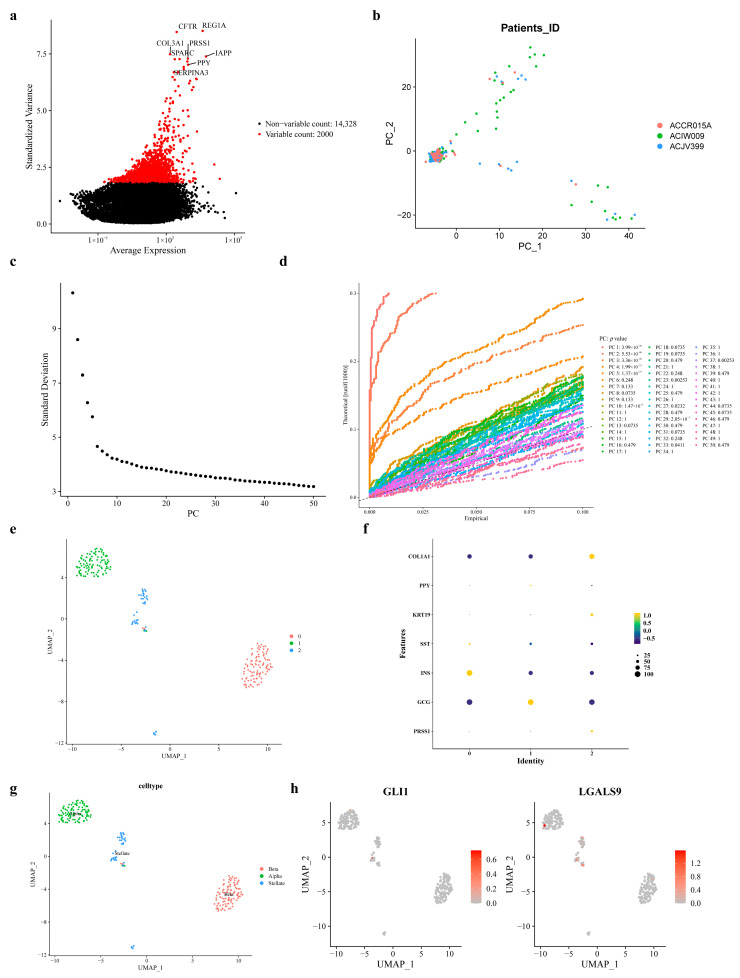
Single-cell data analysis and identification of key cell types. (**a**) Identification of highly variable genes in single-cell RNA sequencing (scRNA-seq) data in the GSE86469 single-cell RNA sequencing dataset. Red represents highly variable genes and black represents non-highly variable genes. (**b**) Principal component analysis (PCA) dimensionality reduction. (**c**) Scree plot. (**d**) Selection of optimal dimensions using the JackStraw method. Different colors represent different principal components. (**e**) UMAP clustering of all sample cells (*n* = 258), where cells are divided into three distinct subpopulations. (**f**) Bubble plot showing the expression of marker genes. Larger and yellower dots indicate higher expression levels. (**g**) Annotation map of the three cell subpopulations. (**h**) Expression distribution of biomarkers across the three cell types. Redder colors indicate higher expression levels. Abbreviations: PCA, principal component analysis; UMAP, uniform manifold approximation and projection; scRNA-seq, single-cell RNA sequencing.

**Figure 8:**
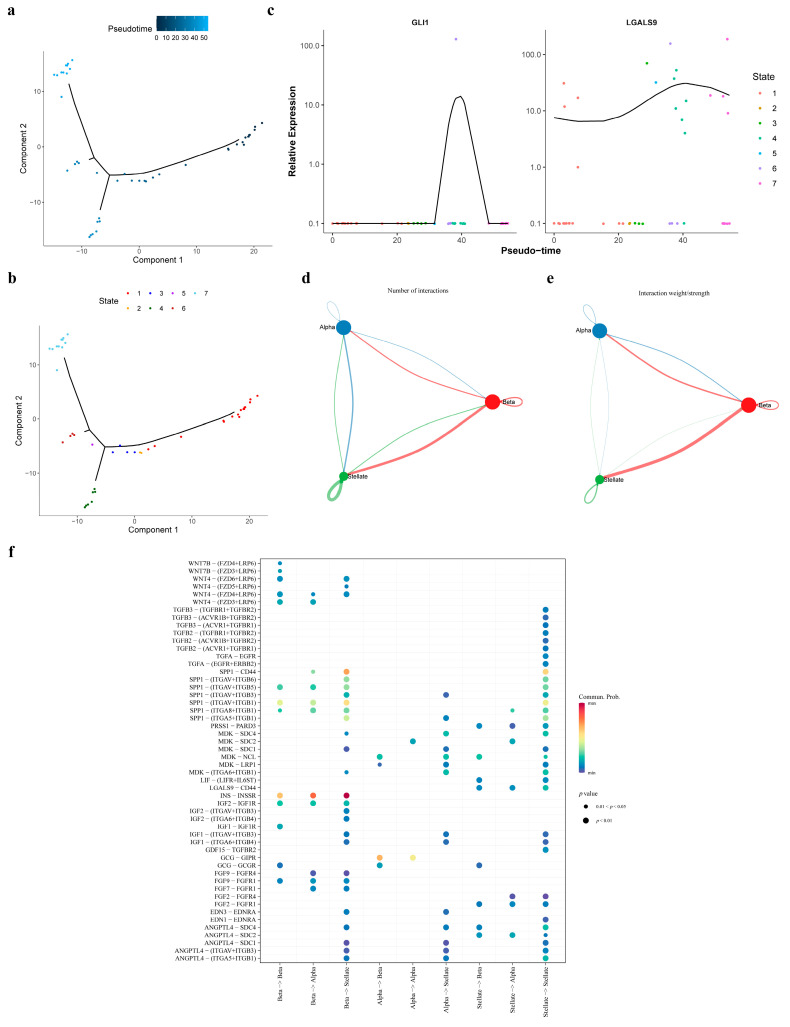
Pseudotime analysis of stellate cells and cell communication. (**a**) Differentiation trajectory of stellate cells based on pseudotime from the GSE86469 dataset. Dark blue indicates early stages of differentiation, while light blue indicates later stages. (**b**) Differentiation states of stellate cells based on pseudotime, revealing a total of six distinct states. (**c**) Dynamic expression of biomarkers along pseudotime. Different colors represent different states. (**d**) Network diagram of cell communication quantity. (**e**) Network diagram of cell communication strength. (**f**) Bubble plot of receptor-ligand communication. Color indicates communication probability, and bubble size indicates the significance level (*p* < 0.01).

After conducting the steps of data filtration and quality control, a total of 258 cells and 16,328 genes were successfully preserved in the GSE86469 dataset (Figure S6). Then, the top 2000 genes exhibiting high variability were identified, of which the top 10 were labeled (Figure 7a). The PCA revealed no statistically significant outliers. The elbow plot indicated a gradual stabilization of the first 10 PCs, thus selecting them for subsequent analysis (Figure 7b–d). Subsequently, 3 clusters were obtained by cell cluster analysis and were annotated, including Alpha, Beta, and Stellate cells (Figure 7e–g, Table S6). It should be noted that the limited cell number and restricted cell-type diversity may reduce resolution and limit fine-grained biological interpretation. Both biomarkers exhibited substantially increased expression within stellate cells, identified as critical cellular components (Figure 7h and Figure S6). The pseudotime analysis of the Stellate cell revealed that the expression levels of *GLI1* and *LGALS9* exhibited temporal changes during differentiation, with a significant increase observed at 40 pseudotime, followed by a subsequent decrease (Figure 8a–c). Moreover, cell communication analysis revealed stronger cellular interactions between Beta cells and with a greater number of ligand-receptor pairs (Figure 8d–f).

## 4. Discussion

Although current therapeutic strategies for T2D are effective in controlling blood glucose levels, they remain insufficient to address disease heterogeneity and multisystem complications, highlighting an urgent need to identify novel therapeutic targets. DOP, as bioactive components derived from a traditional medicinal plant, have been shown to exert antihyperglycemic effects through multiple pathways, including modulation of insulin signaling, suppression of inflammatory responses, and improvement of gut microbiota composition; however, their core target genes and causal relationships with T2D have not yet been fully elucidated [8]. Here, we integrated transcriptomic profiling, network medicine, and Mendelian randomization analyses to identify DOP-associated molecular targets in T2D. Among candidate genes, *GLI1* and *LGALS9* showed associations with T2D and consistent diagnostic performance, whereas *FGF2* failed to meet validation criteria. Multi-omic analyses further suggested that *GLI1* and *LGALS9* may contribute to T2D progression through immune regulation, cellular communication, and microenvironmental remodeling. These findings provide potential molecular insights into DOP-mediated T2D regulation and identify candidate targets for further investigation.

*GLI1*, a Hedgehog signaling effector, has been implicated in metabolic regulation and stromal remodeling beyond its classical developmental functions [38]. The MR analysis in this study indicated that *GLI1* was associated with an odds ratio (OR) of less than 1, suggesting that it may function as a protective factor against type 2 diabetes (T2D). However, *GLI1* expression levels were significantly elevated in patients with T2D in both the discovery and validation cohorts. To further explore this discrepancy, *GLI1* expression was correlated with insulin signaling genes in GSE164416. *GLI1* showed positive correlations with *IRS1* and *PIK3R1*, suggesting that *GLI1* upregulation in T2D may reflect an adaptive transcriptional response associated with altered insulin signaling rather than a pathogenic effect. This finding is consistent with the MR-derived protective association, which reflects genetically predicted *GLI1* effects under basal conditions. However, these associations remain hypothesis-generating and require functional validation. GSEA linked *GLI1* to ECM-related pathways, consistent with the reported role of Hedgehog–GLI signaling in stromal activation and matrix remodeling [39,40]. Our findings suggest that *GLI1* functions as a regulatory node through which DOP may modulate tissue remodeling in T2D. Molecular docking further suggested potential structural interactions between D-galacturonic acid, a monosaccharide component associated with DOP structure, and the *GLI1* protein, with binding energies lower than −5 kcal/mol. Notably, D-galacturonic acid, a characteristic unit of acidic polysaccharides, may facilitate stable interactions with *GLI1* [41]. Given that monosaccharides represent structural components of DOP [42], these predicted interactions provide potential molecular hypotheses for further investigation.

*LGALS9*, encoding Galectin-9, an immune-regulatory lectin involved in inflammatory and metabolic processes [43,44]. MR analysis and expression validation supported *LGALS9* as a potential risk-associated biomarker for T2D. Given its immunomodulatory functions, *LGALS9* may link immune activation with metabolic dysfunction in T2D. While *LGALS9* has been reported in diverse immune-related diseases and may function as a disease-activity marker in certain contexts [45,46], its disease-specific interpretation requires careful contextualization. Molecular docking further suggested a potential molecular basis by which *Dendrobium officinale* polysaccharides (DOP) may regulate *LGALS9*: the DOP-associated monosaccharides D-mannose and L-rhamnose monohydrate both exhibited favorable binding affinities to the *LGALS9* protein, with binding energies below −5 kcal/mol. As *LGALS9* recognizes β-galactoside structures via its carbohydrate recognition domain (CRD) [43,47]. These interactions could alter *LGALS9*-mediated immune responses, suggesting that DOP may alleviate T2D inflammation and metabolic dysregulation through its monosaccharide components. Nonetheless, this hypothesis requires further experimental validation.

Our results align with the recognition that T2D pathogenesis involves immune regulation and microenvironment remodeling [48]. While *LGALS9* and *GLI1* have been studied in immune-related diseases and tissue remodeling [39,40,45,46], their specific roles in T2D remain underexplored. Our integrated multi-omic evidence supports further investigation of these biomarkers in T2D and DOP-based interventions.

Chronic inflammation and immune dysregulation contribute to insulin resistance and β-cell dysfunction in T2D [14,48]. In this study, immune infiltration analysis revealed substantial remodeling of the T2D immune microenvironment, and *GLI1* and *LGALS9* showed distinct associations with immune cell signatures. These findings suggest that these two biomarkers may be associated with transcriptional signatures of immune dysregulation in T2D islets, though causal relationships with specific immune cell populations remain to be confirmed by direct immunophenotyping methods such as flow cytometry or spatial transcriptomics. This differential correlation pattern suggests that the two biomarkers may coordinately contribute to immune imbalance in T2D by regulating distinct arms of the immune system, namely innate and adaptive immunity [49]. *LGALS9* may suppress NK cell cytotoxicity [50], while *GLI1* may influence adaptive immunity by regulating CD4^+^ T cell memory differentiation [51]. These two parallel yet interconnected regulatory axes together shape a chronic inflammatory microenvironment that promotes insulin resistance and pancreatic β-cell dysfunction [14]. Pathway enrichment analyses further revealed a close association between *GLI1* and ECM–receptor interactions, suggesting that immune cell functional states may be modulated by the matrix microenvironment. Under metabolic stress conditions, *GLI1*-mediated ECM remodeling may alter immune cell recruitment, retention, and activation signals—particularly for memory T cells—whereas *LGALS9*, as a key mediator of intercellular communication, may further regulate interactions between NK cells and other immune populations [52]. Disruption of this immune–matrix interaction network may represent a critical step in the transition of T2D from a metabolic disorder to a systemic inflammatory disease. Our findings support a model in which DOP may modulate *GLI1* and *LGALS9* through monosaccharide components, regulating innate immunity, adaptive immunity, and matrix-associated pathways beyond classical glucose metabolism.

Single-cell RNA sequencing analysis provided preliminary evidence suggesting preferential expression of *GLI1* and *LGALS9* in stellate cells within this limited dataset. Accumulating evidence indicates that pancreatic stellate cells (PSCs) actively participate in the pathological progression of metabolic diseases by regulating extracellular matrix (ECM) deposition, paracrine signaling, and microenvironmental inflammatory responses, and that their activation status can directly influence islet architecture and β-cell function [53,54,55]. This cell-type localization integrates the mechanisms described above: *GLI1* may affect immune cell recruitment through the regulation of ECM remodeling, whereas *LGALS9* may modulate the function of surrounding immune cells (such as NK cells) via paracrine signaling [56]. Pseudotime trajectory analysis further revealed that *GLI1* and *LGALS9* expression increased markedly around pseudotime 40 and subsequently declined. This “rise-and-fall” pattern may indicate an early compensatory upregulation in response to metabolic stress and inflammatory stimuli, followed by downregulation due to cellular functional exhaustion or feedback inhibition upon sustained activation [57]. Such dynamic expression features suggest that these two genes may tentatively suggest associations with stellate cell state transitions, though these pseudotime trajectories are derived from a small cell population and require validation in larger, higher-resolution single-cell datasets. Cell–cell communication analysis suggested extensive ligand–receptor interactions between β cells and stellate cells, implying that stellate cells may directly influence β-cell function and survival through the secretion of cytokines, growth factors, or ECM components [58]. This provides a new perspective on the multicellular cooperative pathology of T2D, indicating that β-cell dysfunction arises not only from intrinsic metabolic stress but is also constrained by state changes in stellate cells within the microenvironment. Based on these findings, *Dendrobium officinale* polysaccharides may act on *GLI1* and *LGALS9* within stellate cells through their monosaccharide components, modulating stellate cell activation and thereby exerting systemic metabolic protective effects via coordinated mechanisms involving ECM microenvironment remodeling, immune cell recruitment, and β-cell communication. This stellate cell–centered mode of action offers a cellular biological basis for the multitarget regulatory properties of *Dendrobium officinale* polysaccharides.

This study employed a reverse pharmacology strategy to identify DOP-associated molecular targets in T2D. Integrative analyses identified *GLI1* and *LGALS9* as key candidate biomarkers with genetic associations with T2D. Our findings suggest that DOP may exert regulatory effects through pathways related to immune modulation and microenvironmental remodeling. These results provide potential molecular insights into DOP-mediated T2D regulation and support further experimental validation of these targets. Several limitations should be considered when interpreting the present findings. This study was primarily based on analyses of publicly available datasets combined with bioinformatic inference. Although Mendelian randomization provides a strengthened framework for causal assessment, it does not replace experimental validation in relevant cellular and animal models, which will be necessary to clarify the underlying biological mechanisms. Additionally, immune infiltration was computationally inferred and represents relative enrichment rather than direct immunophenotyping. Nevertheless, the consistency observed across immune cell differences, biomarker-immune correlations, and stellate cell-specific expression supports the internal plausibility of the immune-microenvironment framework proposed in this study. Additionally, ROC analyses in two external cohorts (GSE20966 and GSE38642) showed modest AUCs with wide 95% CIs for *GLI1* and *LGALS9*, suggesting limited generalizability across datasets with different sizes and compositions. Finally, molecular docking only evaluates theoretical structural compatibility and cannot determine binding kinetics, cellular uptake, bioavailability, or downstream biological effects. Moreover, the in vivo metabolism and degradation of DOP-derived monosaccharides were not assessed; therefore, these predicted interactions should be considered hypothesis-generating rather than direct evidence of DOP regulation. Further work will be required to delineate which structural fractions, as well as their bioavailability profiles, are most relevant to *GLI1*- and *LGALS9*-associated pathways.

## Figures and Tables

**Table 1:** Molecular docking binding energy.

Gene	Molecule	PBD ID/PubChem CID	Vina Score
*GLI1*	D-Galacturonic acid	3LSD	−6.1
*LGALS9*	glucose	7T91	−4.8
*LGALS9*	D-Mannose	7T91	−5.5
*LGALS9*	Galactose	7T91	−5
*LGALS9*	L-rhamnose monohydrate	7T91	−5.6
*GLI1*	GANT61 (positive control)	9790140	−5.2

## Data Availability

The data presented in this study are available in the Gene Expression Omnibus (GEO) repository at http://www.ncbi.nlm.nih.gov/geo/, accessed on 6 December 2023, under accession numbers GSE164416, GSE25724, and GSE86469. These data were derived from the following resources available in the public domain: the IEU OpenGWAS database (https://gwas.mrcieu.ac.uk/; accessed on 6 December 2023).

## Supplementary Materials

The following supporting information can be downloaded at: https://www.mdpi.com/article/10.3390/cimb48070672/s1.

## Abbreviations

The following abbreviations are used in this manuscript:

DOP	*Dendrobium officinale* Polysaccharide
T2D	Type 2 Diabetes Mellitus
MR	Mendelian Randomization
ROC	Receiver Operating Characteristic
DCA	Decision Curve Analysis
GEO	Gene Expression Omnibus
GPL	Gene Expression Platform
ENSEMBL	European Molecular Biology Laboratory—European Bioinformatics Institute
DESeq2	Differential Expression Analysis Based on the Negative Binomial Distribution 2
GWAS	Genome-Wide Association Study
eQTL	Expression Quantitative Trait Locus
IEU	Integrative Epidemiology Unit
IVs	Instrumental Variables
SNPs	Single Nucleotide Polymorphisms
LD	Linkage Disequilibrium
IVW	Inverse Variance Weighted
LOO	Leave-One-Out
AUC	Area Under the Curve
GSEA	Gene Set Enrichment Analysis
GO	Gene Ontology
KEGG	Kyoto Encyclopedia of Genes and Genomes
PPI	Protein–Protein Interaction
ssGSEA	Single Sample Gene Set Enrichment Analysis
GSVA	Gene Set Variation Analysis
TF	Transcription Factor
miRNA	MicroRNA
PDB	Protein Data Bank
scRNA-seq	Single-Cell RNA Sequencing
PCA	Principal Component Analysis
UMAP	Uniform Manifold Approximation and Projection
ECM	Extracellular Matrix
CRD	Carbohydrate Recognition Domain
PSCs	Pancreatic Stellate Cells
