# Supplementary material for: Identification of Biomarkers for Dendrobium officinale Polysaccharide in Type 2 Diabetes Mellitus via Integrated Network Pharmacology and Mendelian Randomization
Source: Curr Issues Mol Biol. 2026 Jun 29;48(7):672. doi: 10.3390/cimb48070672 (PMC13407339; doi:10.3390/cimb48070672)
Supplement: Supplementary file 1 [file cimb-48-00672-s001.zip › cimb-4362046-supplementary materials-done.pdf]

current issues in molecular biology

MDPI

Supplementary Information

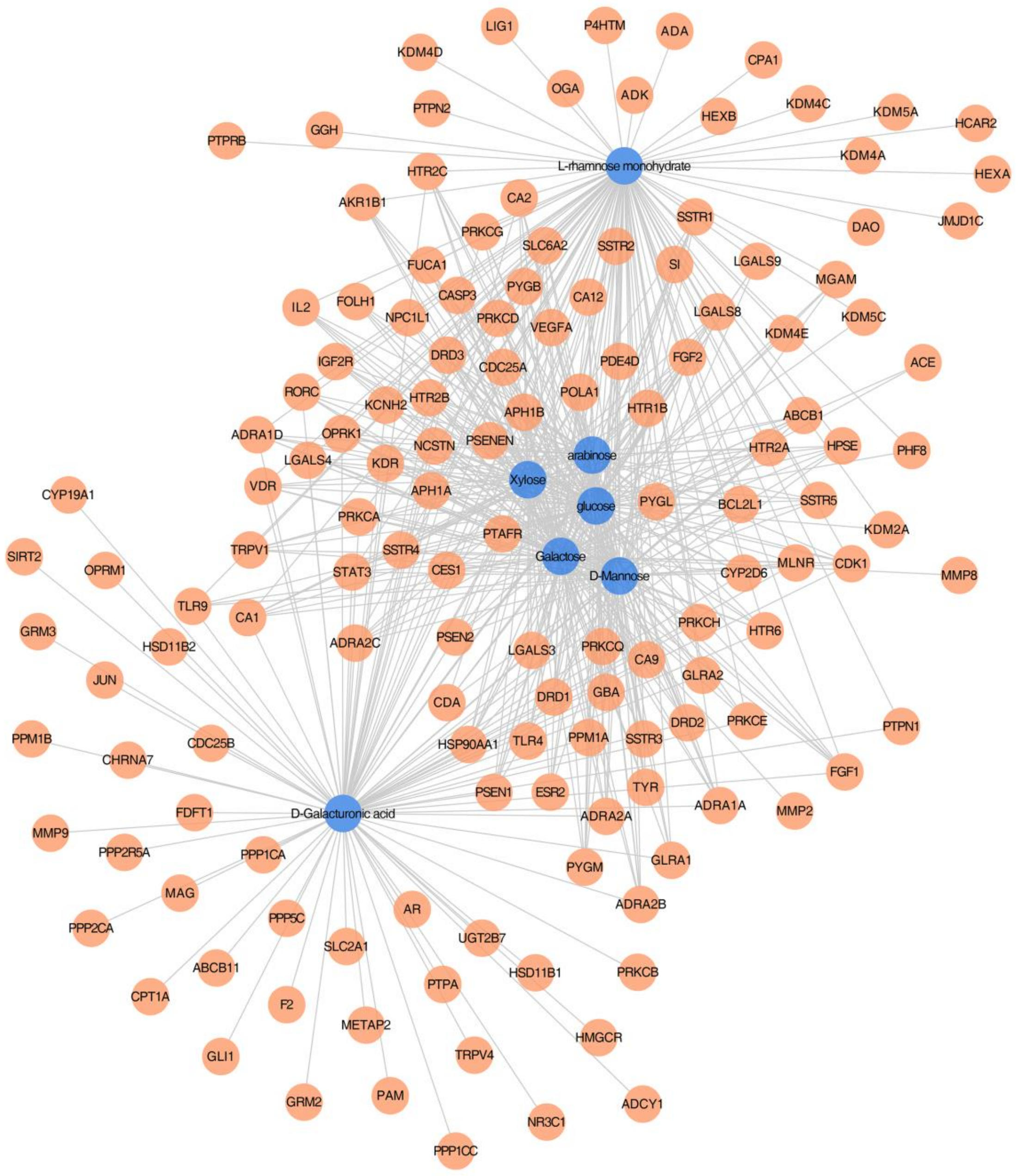

Figure S1: Monosaccharide-target gene network diagram. Blue represents compounds and orange represents target genes

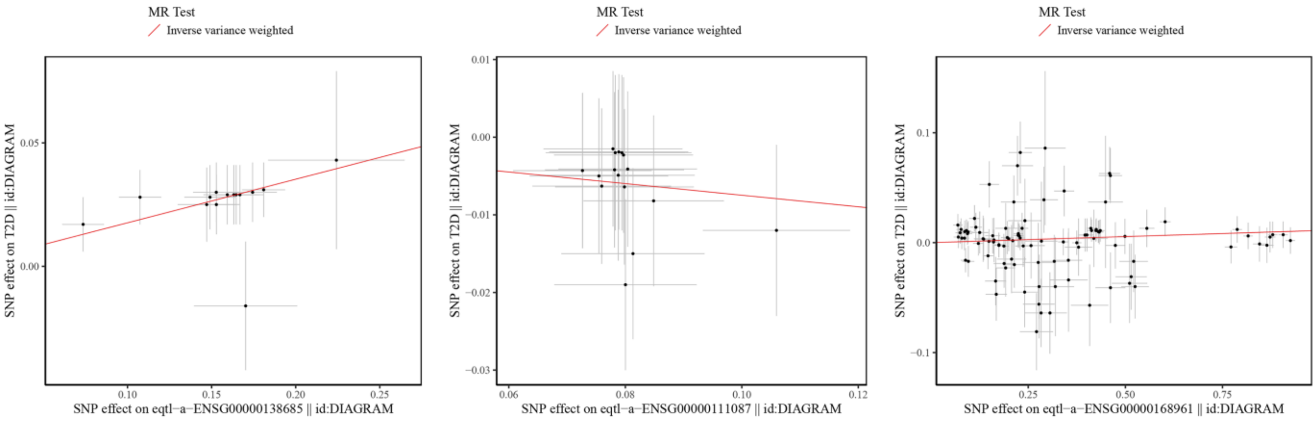

Figure S2: Mendelian randomization (MR) scatter plot, showing the correlation between the exposure factors and the outcome

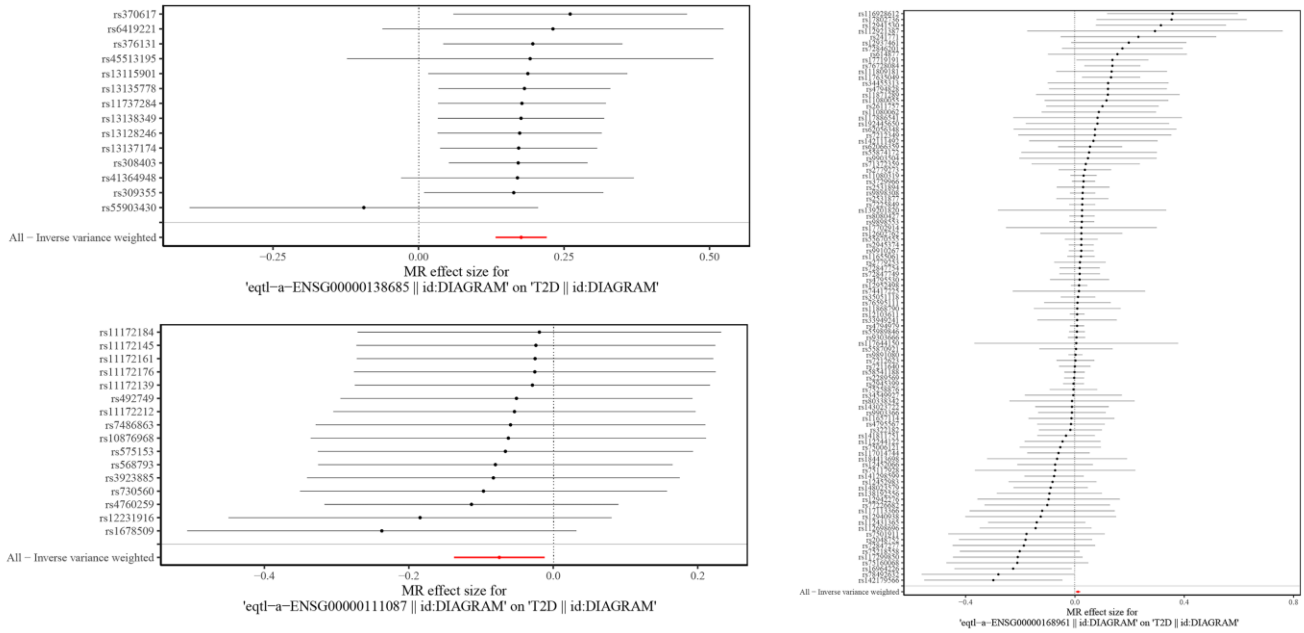

Figure S3: Mendelian randomization (MR) forest plot. Data on the right side suggest risk factors, while data on the left side suggest protective factors

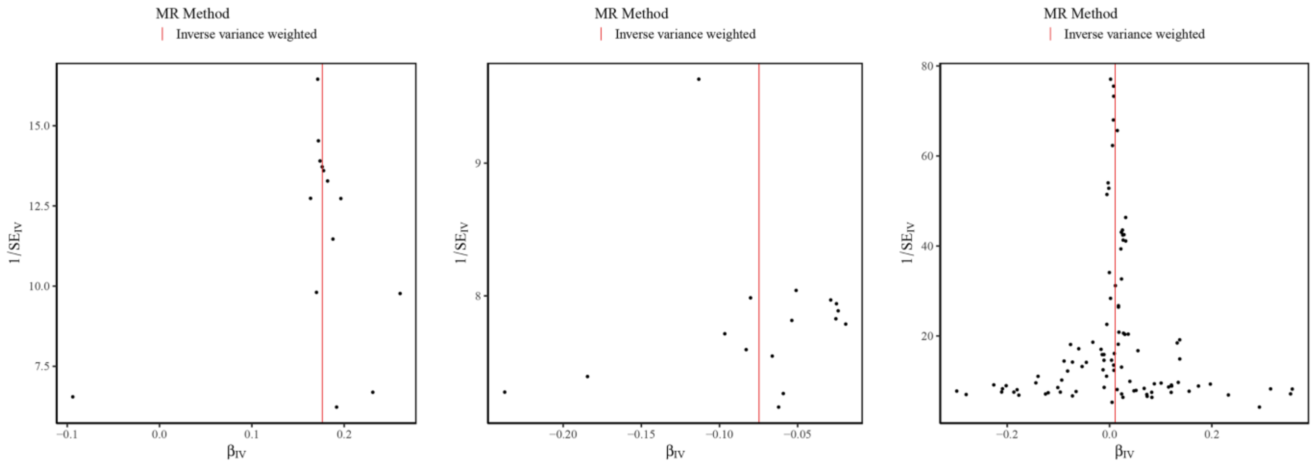

Figure S4: Mendelian randomization (MR) funnel plot. The distributions of the three genes are relatively symmetrical, consistent with Mendel's second law

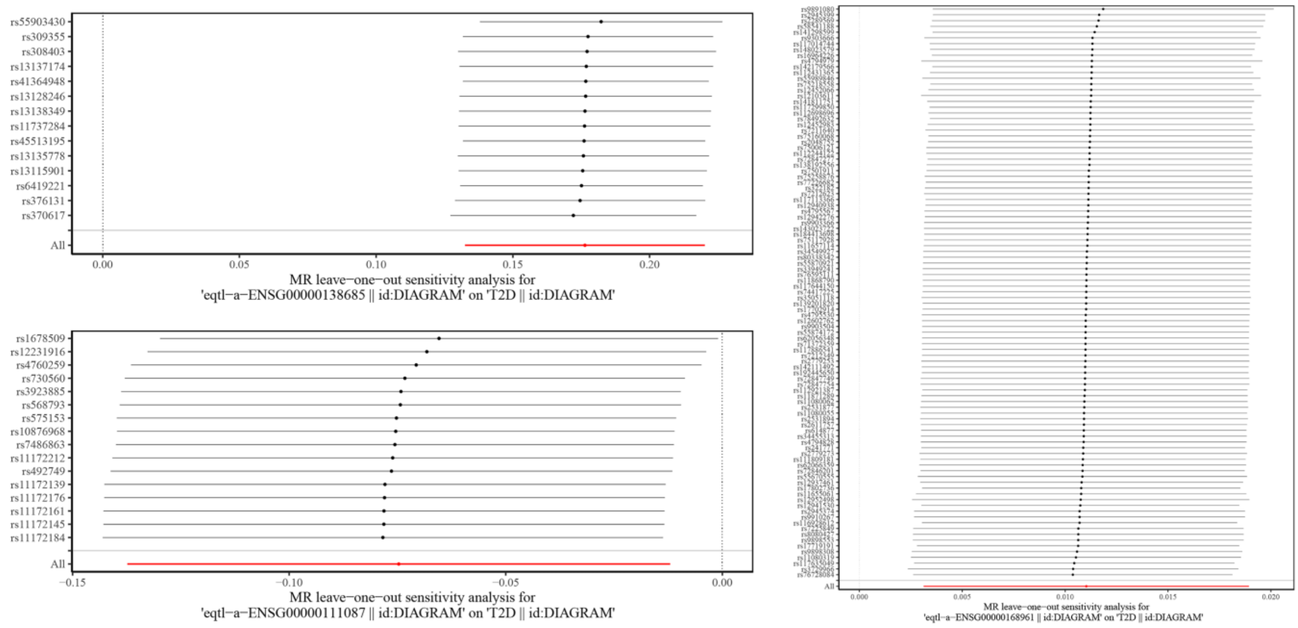

Figure S5: Sensitivity analysis results. The figure illustrates the validation of the robustness of the Mendelian randomization (MR) results through leave-one-out (LOO) analysis, showing the changes and consistency in the overall effect estimates after removing each single nucleotide polymorphism (SNP) one by one

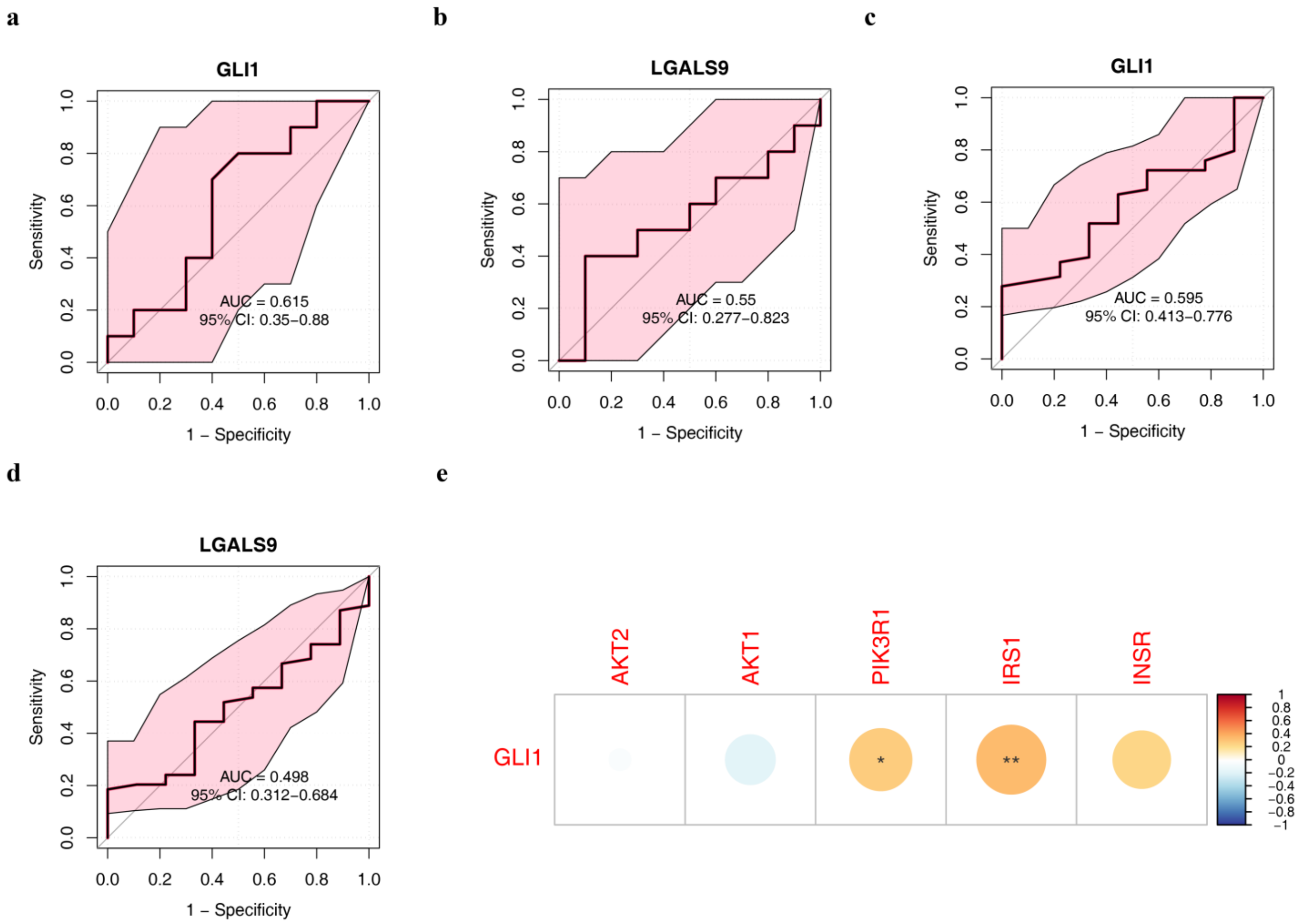

Figure S6: External validation of diagnostic performance and correlation analysis of GLI1 with insulin signaling genes. (a–d) ROC curves of GLI1 and LGALS9 in GSE20966 and GSE38642 co-horts. AUC values with 95% confidence intervals were calculated to assess diagnostic perfor-mance. (e) Spearman correlation analysis between GLI1 and insulin signal-ing genes (AKT2, AKT1, PIK3R1, IRS1, and INSR) in GSE164416. Circle size and color indicate correlation strength and direction, respectively. *$p < 0.05$; **$p < 0.01$.

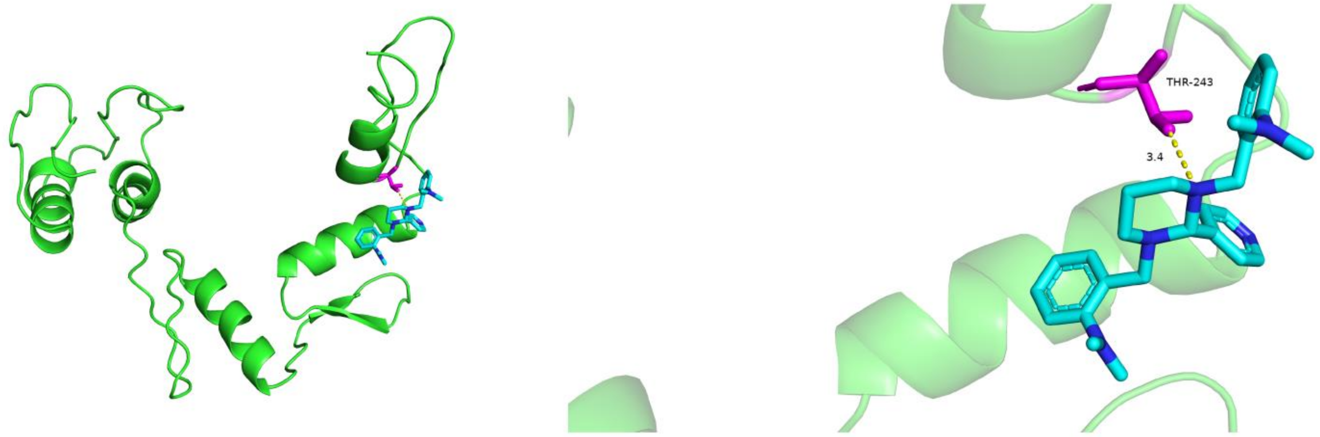

Figure S7: Validation of molecular docking protocol. GANT61 (a GLI1 inhibitor) was docked to GLI1 as a positive control, yielding a binding energy of −5.2 kcal/mol

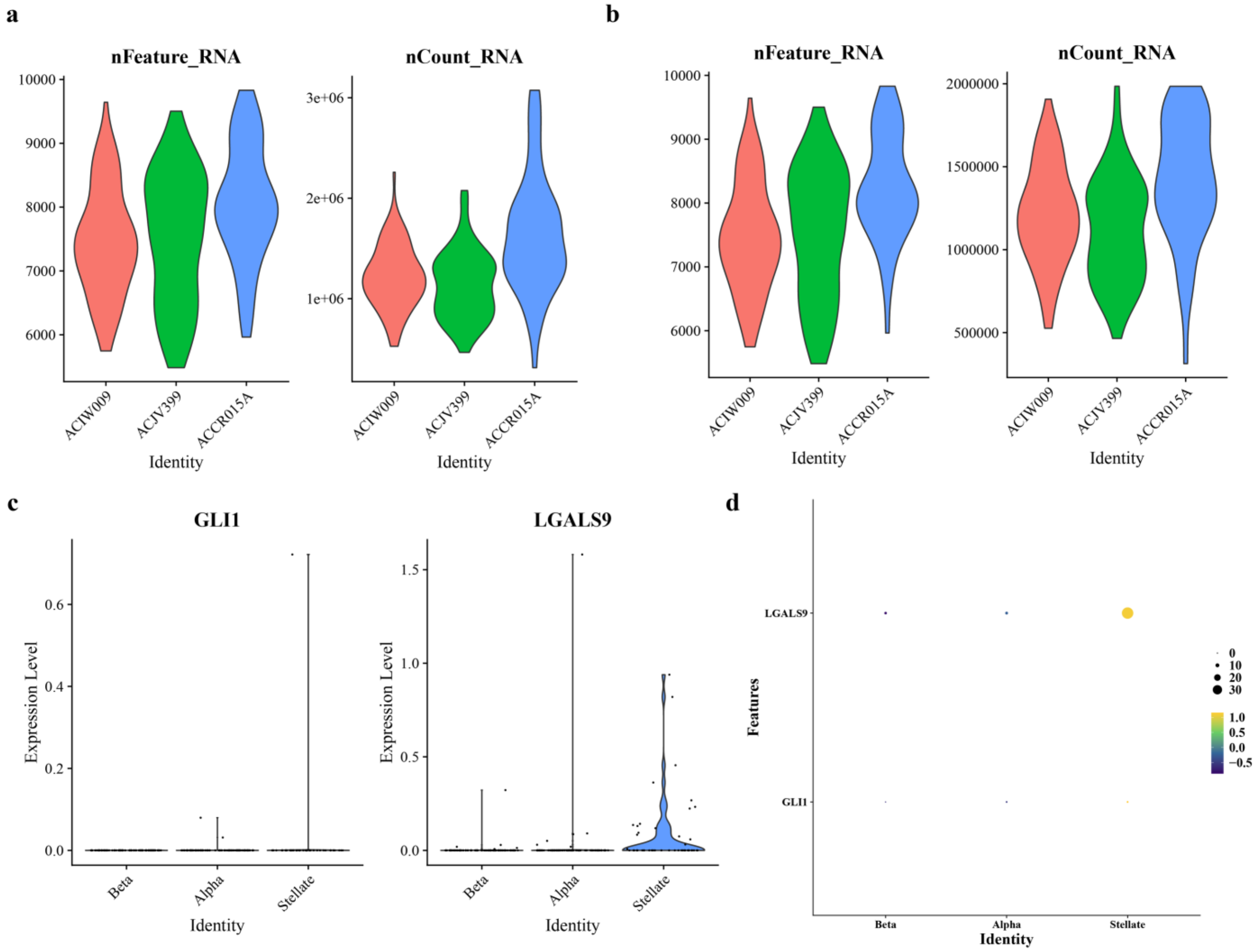

Figure S8: Quality control of single-cell data and validation of biomarker expression characteristics. (a) Distribution plots of nFeature_RNA and nCount_RNA before quality control. (b) Distribution plots of nFeature_RNA and nCount_RNA after quality control. (c) Violin plots showing biomarker expression across different cell types. (d) Bubble plot showing biomarker expression across different cell types
